# Supplementary material for: The Evolution and Characterization of the RNA Interference Pathways in Lophotrochozoa
Source: Genome Biol Evol. 2024 May 7;16(5):evae098. doi: 10.1093/gbe/evae098 (PMC11114477; doi:10.1093/gbe/evae098)
Supplement: evae098_Supplementary_Data [file evae098_supplementary_data.zip › Supplementary_materials.zip!/selected_miRNAs/CrGi_selected_miRNAs/CrGi_NC_047568.1_32754.pdf]

Provisional ID	:	NC_047568.1_32754
Score total	:	690.9
Score for star read(s)	:	3.9
Score for read counts	:	691.4
Score for mfe	:	-1.5
Score for randfold	:	-2.2
Score for cons. seed	:	-0.6
Total read count	:	1368
Mature read count	:	1340
Loop read count	:	0
Star read count	:	28

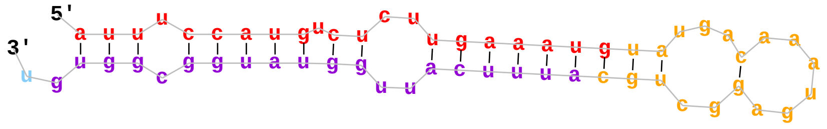

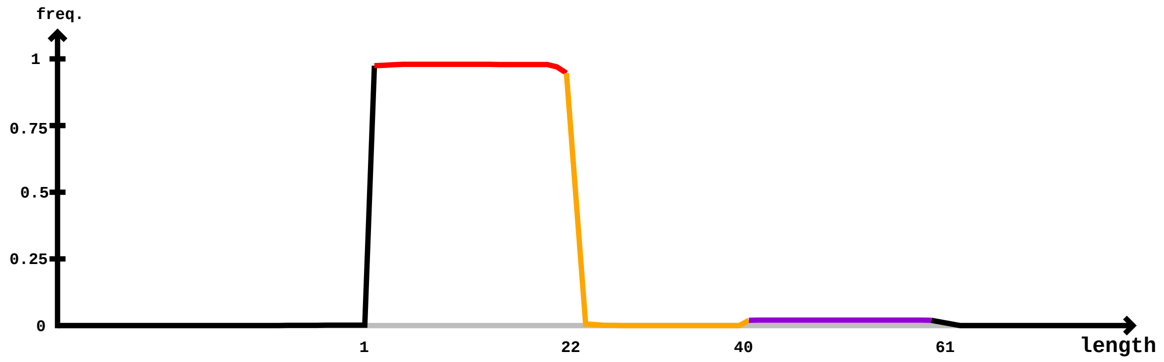


```
                                      Mature                                   Star
5'- uuccaucaacaguuccugauauuagagcaauauuuccaugucucuugaaauguaugacaaaugaggcugcauuucauugguauggcgguguuguccguuuuugcugcauu  -3'   obs
    uuccaucaacaguuccugauauuagagcaauauuuccaugucucuugaaauguaugacaaaugaggcugcauuucauugguauggcgguguuguccguuuuugcugcauu         exp
    .........((((....(((....(.((((((((.(((((.((..(((((((((...(......)..)))))))))..))))))).)))))))).))))...))))....  reads   mm   sample
    ...............................auuuccaugucucuugaaa............................................................  3       0    Mt2
    ...............................auuuccaugucucuugaaau...........................................................  6       0    Mt2
    ...............................auuuccaugCcucuugaaau...........................................................  1       1    Mt2
    ...............................auuuccaugucucuugUaaug..........................................................  1       1    Mt2
    ...............................auuuccaugCcucuugaaaug..........................................................  57      1    Mt2
    ...............................auuCccaugucucuugaaaug..........................................................  1       1    Mt2
    ...............................auuuccaugucucuugaGaug..........................................................  1       1    Mt2
    ...............................auuuccaugucucuugaaaug..........................................................  329     0    Mt2
    ...............................auuuccaugucucuugaaaugC.........................................................  1       1    Mt2
    ...............................auuuccaugucucuugaaaugu.........................................................  24      0    Mt2
    ...............................auuuccaugucucuugaaaugua........................................................  1       0    Mt2
    ...............................auuuccaugucucuugaaauguau.......................................................  1       0    Mt2
    ..................................uccaugucucuugaaaug..........................................................  1       0    Mt2
    ......................................................................auuucauugguauggUggug....................  2       1    Mt2
    ......................................................................auuucauugguauggcggug....................  7       0    Mt2
    ......................................................................auuucauugguauggcgguguu..................  1       0    Mt2
    ......................................................................auuucauugguauggUggugu...................  1       1    Mt2
    ......................................................................auuucauugguauggcgguguu..................  2       0    Mt2

    ...............................auuuccaugucucuugaaa............................................................  5       0    Mt1
    ...............................auuuccaugCcucuugaaau...........................................................  1       1    Mt1
    ...............................auuuccaugucucuugaaau...........................................................  10      0    Mt1
    ...............................auuuccaugucucuugUaaug..........................................................  1       1    Mt1
    ...............................auuuccaugucucAugaaaug..........................................................  1       1    Mt1
    ...............................auuuccaugucucuugaaaug..........................................................  269     0    Mt1
    ...............................auuuccaugCcucuugaaaug..........................................................  49      1    Mt1
    ...............................auCuccaugucucuugaaaug..........................................................  1       1    Mt1
    ...............................auuuccaugucucuugaaaugu.........................................................  10      0    Mt1
    ...............................auuuccaugCcucuugaaaugu.........................................................  1       1    Mt1
    ...............................auuuccaugucucuugaaaugua........................................................  1       0    Mt1
    ...............................auuuccaugucucuugaaauguau.......................................................  1       0    Mt1
    .................................uuccaugucucuugaaaug..........................................................  1       0    Mt1
    ..................................uccaugucucuugaaaug..........................................................  1       0    Mt1
```

```
                                 Mature                                 Star
uuccaucaacaguuccugauauuagagcaauauuuccaugucucuugaaauguaugacaaaugaggcugcauuucauugguauggcgguguuguccguuuuugcugcauu
..................................uccaugucucuugaaaug..........................................................    1     0    Mt1
......................................................................auuucauugguauggcggCg....................    1     1    Mt1
......................................................................auuucauugguauggcggug....................    4     0    Mt1
......................................................................auuucauugguauggcgguguA..................    1     1    Mt1
.......................................................................uuucauugguauggcggugu...................    1     0    Mt1

..........................gcaauauuuccaugucucuugaaa............................................................    1     0    Ov1
...............................auuuccaugucucuugaaa............................................................    2     0    Ov1
...............................auuuccaugucucuugaaau...........................................................    3     0    Ov1
...............................auuuccaugCcucuugaaau...........................................................    3     1    Ov1
...............................auuuccaugucucAugaaaug..........................................................    1     1    Ov1
...............................auuuccaugucucuugaaaug..........................................................    217   0    Ov1
...............................auuuccaugCcucuugaaaug..........................................................    38    1    Ov1
...............................auuuccaugucuAuugaaaug..........................................................    1     1    Ov1
...............................auuuccaugucucuugaaaugu.........................................................    12    0    Ov1
...............................auuuccaugucucuugaaaugua........................................................    1     0    Ov1
................................uuuccaugucucuugaaauguau.......................................................    1     0    Ov1
......................................................................auuucauugguauggcggug....................    2     0    Ov1
......................................................................auuucauugguauggUggug....................    1     1    Ov1

......................uagagcaauauuuccaugucuc..................................................................    1     0    Ov2
...............................auuuccaugucucuugaaa............................................................    1     0    Ov2
...............................auuuccaugucucuugaaau...........................................................    5     0    Ov2
...............................auuuccaugCcucuugaaau...........................................................    3     1    Ov2
...............................auuuccaugucucuugCaaug..........................................................    1     1    Ov2
...............................auuuccaugucucuugaaaug..........................................................    206   0    Ov2
...............................auuuccaugCcucuugaaaug..........................................................    51    1    Ov2
...............................auuuccUugucucuugaaaug..........................................................    1     1    Ov2
...............................auuuccaugCcucuugaaaugu.........................................................    2     1    Ov2
...............................auuuccaugucucuugaaaugu.........................................................    9     0    Ov2
.................................uuccaugucucuugaaauguau.......................................................    1     0    Ov2
.................................uuccaugucucuugaaauguauga.....................................................    1     0    Ov2
......................................................................auuucauugguauggcggu.....................    1     0    Ov2
......................................................................auuucauugguauggcggug....................    4     0    Ov2
```
